# Identification of Key Periodontitis Genes and Their Mechanisms of Action Using Comprehensive Multiple Microarray Analysis and Mendelian Randomization Methods

**DOI:** 10.1155/ijog/5587468

**Published:** 2025-10-23

**Authors:** Kezhen Xiang, Conghua Li, Na Hu

**Affiliations:** ^1^ College of Stomatology, Chongqing Medical University, Chongqing, China, cqmu.edu.cn; ^2^ Chongqing Municipal Key Laboratory of Oral Biomedical Engineering of Higher Education, Chongqing, China; ^3^ Chongqing Municipal Health Commission Key Laboratory of Oral Biomedical Engineering, Chongqing, China; ^4^ Chongqing Key Laboratory of Oral Diseases, Chongqing, China

**Keywords:** CD27, genetic causation, Mendelian randomization, periodontitis, weighted gene coexpression network analysis

## Abstract

**Background:**

Periodontitis, a chronic inflammatory disease, damages the soft tissues and bones around the teeth. Affecting adults, mild periodontitis is common, while severe cases impact up to 20% of individuals, with a prevalence of 45%–50%. This study was aimed at identifying and analyzing the functions of genes differentially expressed in periodontitis through bioinformatics. Additionally, we aimed to validate the causal relationships of these genes with periodontitis using Mendelian randomization.

**Methods:**

The investigation included 557 samples obtained from 210 patients suffering from periodontitis within the GEO database, focusing on the differential expression of genes and conducting a weighted gene coexpression network analysis (WGCNA). Hub genes associated with periodontitis were identified for subsequent functional enrichment and pathway analysis through Gene Ontology (GO) and the Kyoto Encyclopedia of Genes and Genomes (KEGG). The diagnostic performance of predictive models for the five most significant hub genes was assessed using receiver operating characteristic (ROC) curves. Finally, we performed Mendelian randomization analysis to evaluate the genetic causal links between the hub genes and periodontitis.

**Results:**

By intersecting WGCNA’s most relevant module genes with significantly differentially expressed genes, we identified 98 hub genes. GO and KEGG analyses underscored the roles of these hub genes in immune cell activation, cytokine signaling, and inflammation. Cytoscape analysis of the top five hub genes, including CXCR4, CD19, CD27, FCGR3B, and CD79A, was conducted. ROC analysis demonstrated excellent performance of the linear predictor model in predicting the risk of periodontitis. Through the application of the inverse variance weighted (IVW) approach, our analysis revealed that the central gene CD27 is linked to periodontitis (OR = 0.7536, 95*%*CI = 0.5886–0.9647, *p* = 0.02477).

**Conclusion:**

Our analysis established a genetic link between the CD27 gene and periodontitis, indicating its potential as a diagnostic or therapeutic target.

## 1. Introduction

Periodontitis affects the soft tissues and bone structures around the teeth as a chronic inflammatory disease. Prevalent among adults, periodontitis sees mild cases frequently, while severe cases occur in up to 20% of individuals, boasting an overall prevalence of 45%–50% [1, 2]. It ranks as the sixth most prevalent human disease globally, with severe forms affecting 11.2% of the population [3]. The complex etiology of periodontitis stems primarily from the interaction between oral bacterial infections and the host’s immune response, also influenced by individual behavior and systemic health factors [4]. As a widespread chronic infectious disease, periodontitis significantly affects millions of people’s oral health and quality of life. Characterized by inflammatory responses to oral bacteria, periodontitis leads to periodontal pockets, alveolar bone loss, and gingival inflammation, making it a primary cause of adult tooth loss. Recent studies show periodontitis’ impact extends beyond the oral cavity, closely associating with systemic health issues like cardiovascular disease, diabetes, and preterm birth, highlighting its public health significance [5–9].

In recent years, Mendelian randomization (MR) has attracted significant interest. This approach employs single nucleotide polymorphisms (SNPs) as instrumental variables (IVs) to accurately deduce causal links between exposure factors and outcomes, thereby successfully addressing the confounding and reverse causation biases that are typically present in conventional studies [10]. Employing MR methodology based on genetic variation, this study explores potential causal relationships between gene expressions associated with periodontitis, overcoming traditional study limitations [11–13].

Comparing data from periodontitis patients and healthy controls, this study is aimed at identifying DEGs associated with periodontitis. Using WGCNA, we identify genes closely associated with periodontitis, further refining gene selection. We aim to identify hub genes as potential diagnostic biomarkers for periodontitis, offering new insights into its diagnosis and treatment mechanisms. Additionally, MR analysis helps us investigate the causal relationship between CD27 and periodontitis, aiming to offer scientific evidence for future treatment strategies and new preventive and management approaches for periodontitis and its related systemic diseases.

## 2. Materials and Methods

### 2.1. Data Source

The research utilized a dataset related to periodontitis sourced from the Gene Expression Omnibus (GEO). GSE16134 contains 310 periodontal gingival tissue samples from 120 patients, and GSE10334 contains 247 samples from 90 patients, all of which come from multiple sampling sites of each patient (such as gingival sulcular fluid and supragingival and subgingival tissues). Therefore, a total of 557 samples corresponding to cross‐site multipoint data of 210 independent patients was obtained.

### 2.2. Identification of Differentially Expressed Genes

We preprocessed GSE16134 and GSE10334 in R 4.1.1, performing batch‐effect correction and normalization. Differential expression was analyzed with the limma package [14], defining DEGs by |LogFC| > 1 and adjusted *p* < 0.05. Heatmaps and volcano plots were generated using pheatmap and ggplot2.

### 2.3. Weighted Gene Coexpression Network Analysis (WGCNA)

Using the WGCNA package [15], we first determined the soft‐thresholding power (*β*) by evaluating scale‐free topology fit indices from *β* = 1 to 20 and chose *β* = 6 at which the model reached *R* ^2^ > 0.85. A signed coexpression network was then constructed using this power. Genes were hierarchically clustered based on topological overlap, with a minimum module size of 30. Modules with eigengene dissimilarity (1–correlation) less than 0.25 (cutHeight = 0.25) were merged. We calculated Pearson correlations between module eigengenes and periodontitis status and selected the module with the highest |*r*| and *p* < 0.01 for downstream analyses.

### 2.4. Candidate Key Gene Selection and GO/KEGG Analysis

We intersected DEGs with genes from the key WGCNA module to obtain candidate hubs and then performed GO and KEGG enrichment via clusterProfiler [16] to reveal their predominant biological processes and pathways.

### 2.5. Protein–Protein Interaction (PPI) Network Hub Gene Identification

Significant genes were submitted to the STRING database [17] to build a PPI network. We imported the network into Cytoscape and applied the CytoHubba plugin to rank and extract the top 10 hub genes.

### 2.6. Forest Plot Model Construction

A multivariable forest plot of the five most significant hub genes was generated with the rms package [18], and model discrimination was quantified by Harrell’s *C*‐index. Diagnostic performance was further evaluated via ROC curves using pROC [19].

### 2.7. Immune Cell Analysis

We applied CIBERSORT [20] to estimate the relative proportions of 22 immune cell types in each sample and compared infiltration profiles between periodontitis and control groups.

### 2.8. MR

To assess the causal relationship between hub gene expression and periodontitis risk, we performed two‐sample MR using publicly available GWAS summary statistics and the TwoSampleMR R package [21]. Instrumental SNPs were chosen based on genome‐wide significance (*p* < 5 × 10^−8^) and pruned for linkage disequilibrium at *r* ^2^ < 0.01 to minimize confounding by population structure. Our analysis rests on three core assumptions: relevance (SNPs are strongly associated with gene expression), independence (SNPs are uncorrelated with confounders, ensured via LD clumping and original GWAS population‐structure correction), and exclusion restriction (SNPs influence periodontitis risk only through gene expression, with no horizontal pleiotropy). We estimated causal effects primarily by inverse variance weighting (IVW) and conducted sensitivity analyses using MR–Egger regression (including the intercept test for pleiotropy), weighted‐median estimation, and MR‐PRESSO global and outlier tests to detect and correct for invalid instruments. Heterogeneity among SNP estimates was evaluated by Cochran’s *Q* statistic, providing a comprehensive framework to strengthen causal inference.

## 3. Results

### 3.1. DEG Screening

Utilizing periodontitis datasets from the GEO database, GSE16134 (including 120 participants and 310 samples) and GSE10334 (including 90 participants and 247 samples), we identified 143 DEGs in the periodontitis group versus controls, with 115 upregulated and 28 downregulated genes (Figure 1a,b; Supporting Information 1: Table S1).

Figure 1Differentially expressed genes between the periodontitis group and the normal group. (a) Heatmap of the top 50 up‐ and downregulated genes in GSE16134 (*n* = 310 samples from 120 patients) and GSE10334 (*n* = 247 samples from 90 patients) after batch correction and normalization. Rows represent genes (|LogFC| > 1, adj. *p* < 0.05), columns represent samples (periodontitis vs. control), and the color scale indicates *z*‐score–normalized expression. (b) Volcano plot summarizing differential expression: *x*‐axis is Log2 fold change, *y*‐axis is −Log10 (adjusted *p* value). Red dots = upregulated genes (LogFC > 1, adj. *p* < 0.05), blue dots = downregulated genes (LogFC < −1, adj. *p* < 0.05), gray = nonsignificant.

**Figure:**
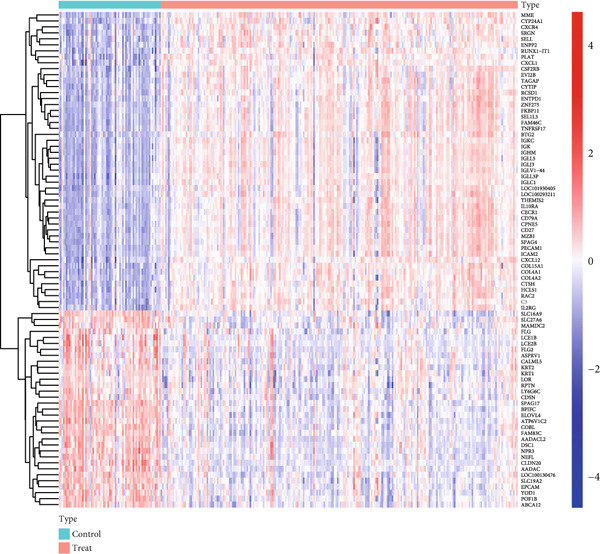
(a)

**Figure:**
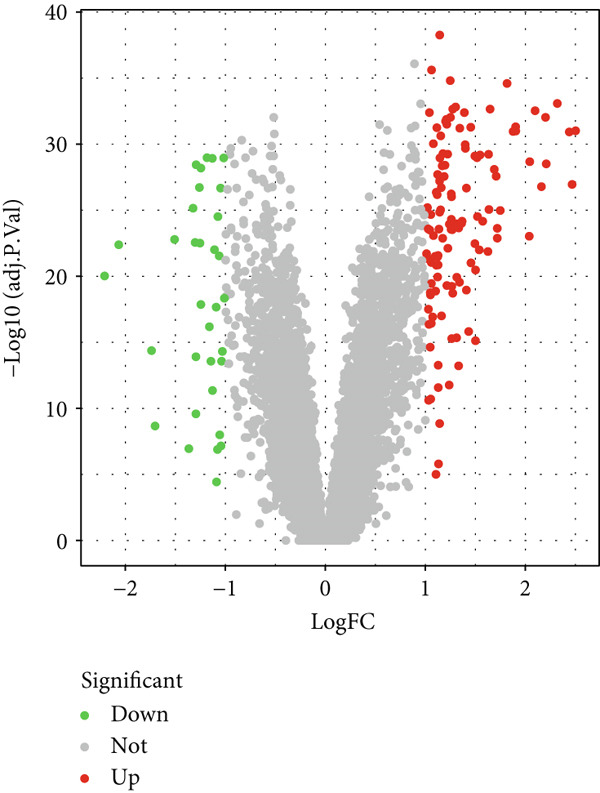
(b)

### 3.2. WGCNA Network Construction and Identification of Periodontitis‐Associated Modules

WGCNA analysis on GSE16134 and GSE10334 datasets identified 12 gene modules, with the “MEblue” module emerging as the most closely associated with periodontitis (Figures 2a, 2b, and 2c; Supporting Information 2: Table S2), highlighting its key role in disease progression.

Figure 2Identification of periodontitis‐associated gene modules using WGCNA in the GEO dataset. (a) Dendrogram of all genes in GSE16134 and GSE10334 clustered based on topological overlap matrix (1‐TOM). (b) Module–trait heatmap showing Pearson correlation coefficients (top) and *p* values (bottom) between each module eigengene and periodontitis status. (c) Scatterplot of gene significance versus module membership in the “blue” module (highest correlation), showing genes most strongly associated with disease.

**Figure:**
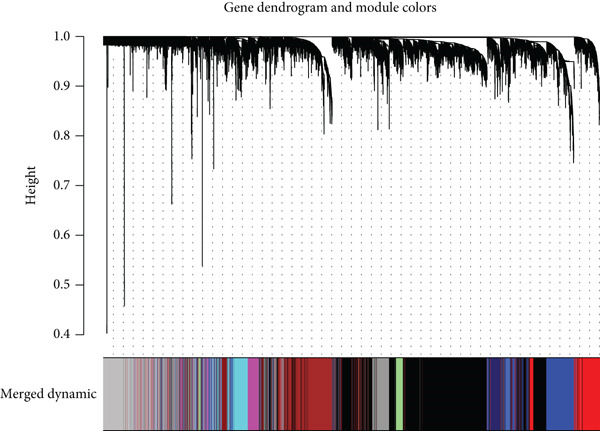
(a)

**Figure:**
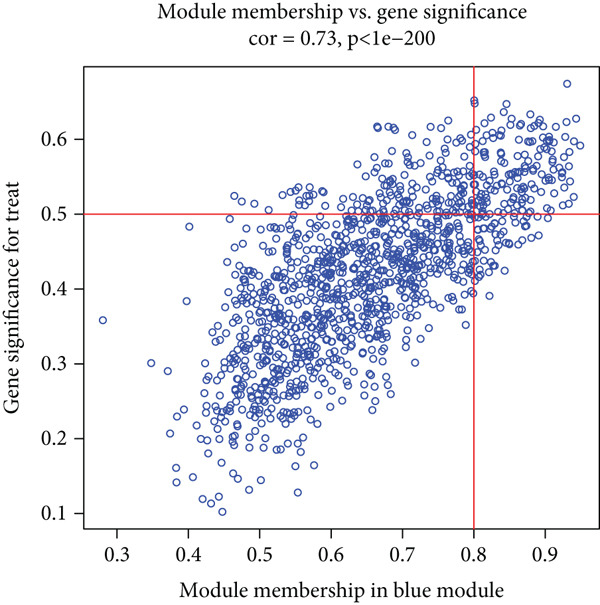
(b)

**Figure:**
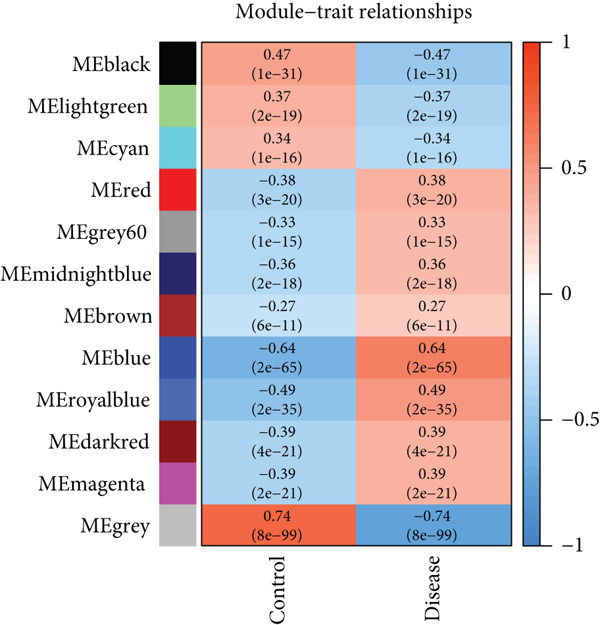
(c)

### 3.3. GO/KEGG Analysis

Cross‐analysis between WGCNA central genes and DEGs uncovered 98 overlapping genes potentially crucial for periodontitis’ development (Figure 3a). GO and KEGG analyses further explored the biological processes and signaling pathways of these genes (Figures 3b, 3c, and 3d).

Figure 3Selection and validation of candidate hub genes. (a) Venn diagram showing 98 overlapping genes between DEGs and “blue” module genes. Numbers indicate gene counts in each set. (b, c) GO enrichment analysis of candidate hub genes. (d) KEGG pathway analysis of candidate hub genes.

**Figure:**
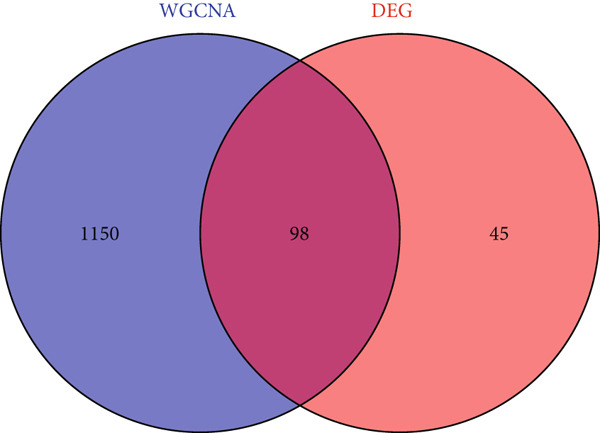
(a)

**Figure:**
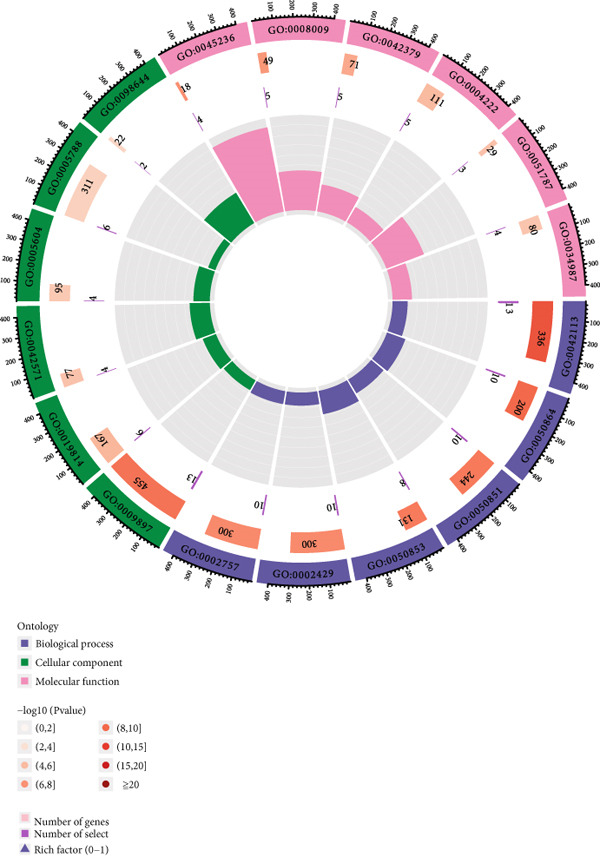
(b)

**Figure:**
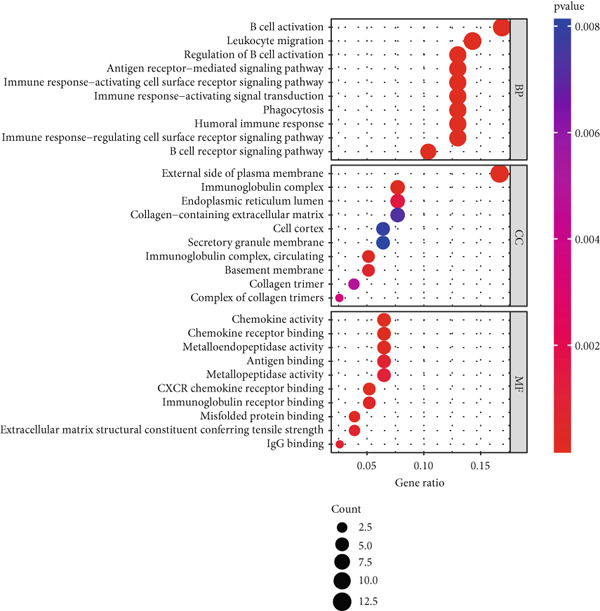
(c)

**Figure:**
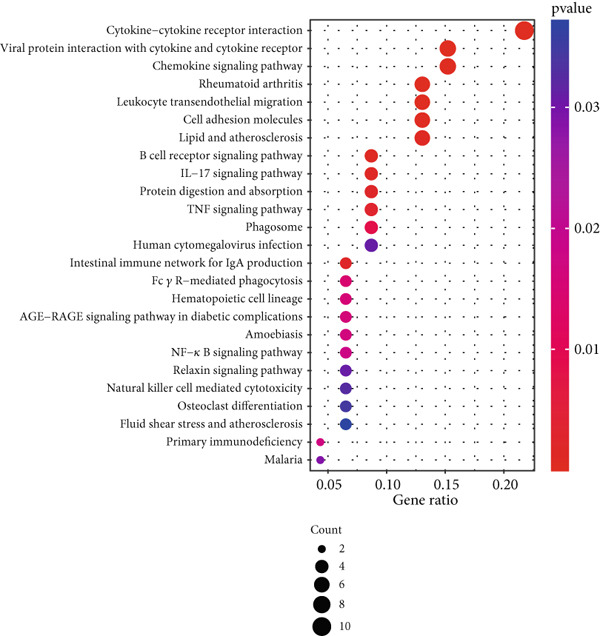
(d)

### 3.4. PPI Network Analysis of Hub Genes

Using the STRING tool, we constructed a PPI network for key genes and visualized it with Cytoscape, focusing on the top 10 ranked genes (including CXCR4, FCGR3B, CXCL13, SELL, CD19, CD38, CD79A, CD27, MME, and PECAM1), highlighting their network centrality (Figure 4a,b).

Figure 4Construction of PPI network. (a) PPI network of overlapping hub genes. (b) CytoHubba‐extracted top 10 hub genes ranked by MCC score, highlighted in red, with MCC values indicated.

**Figure:**
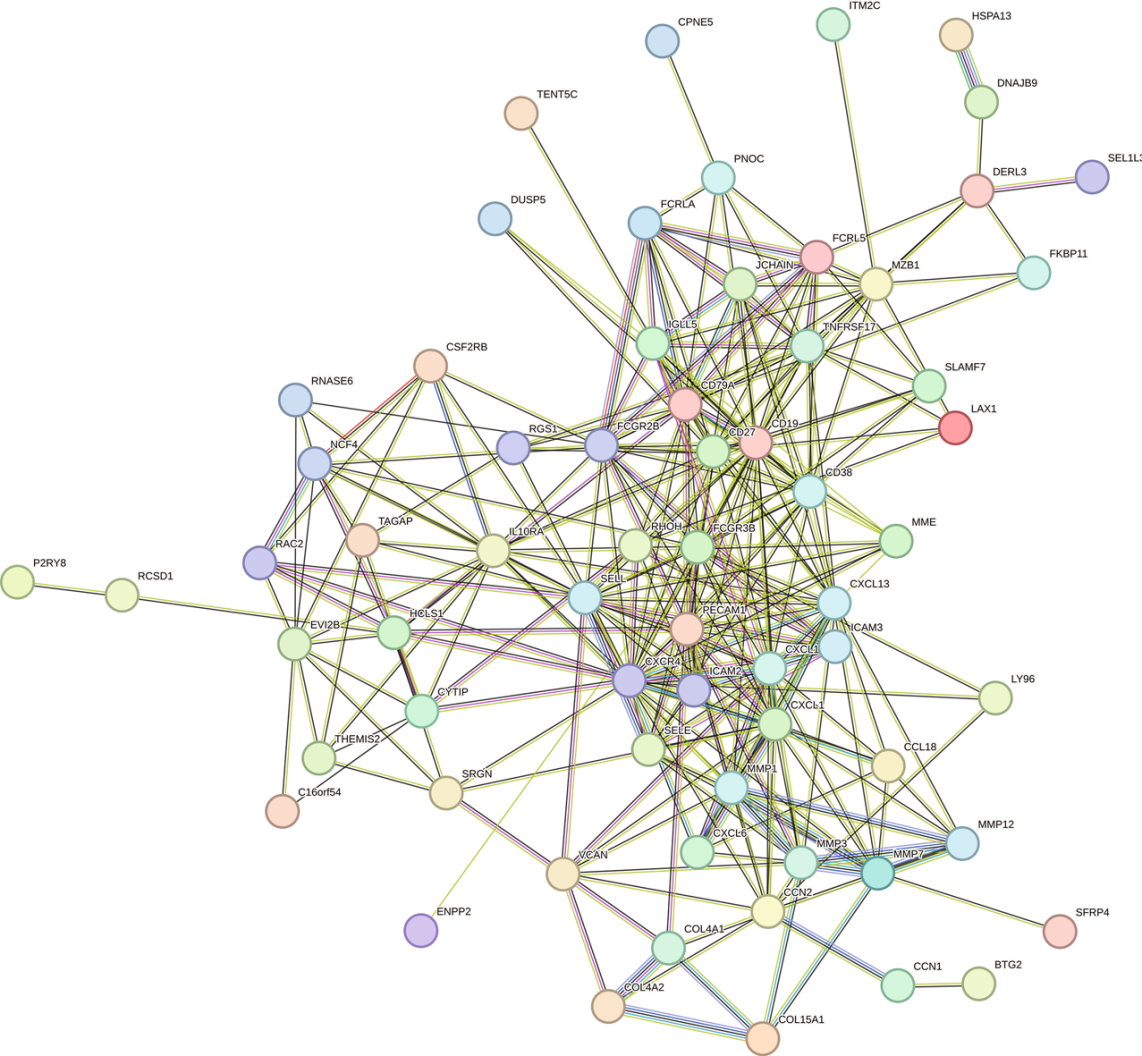
(a)

**Figure:**
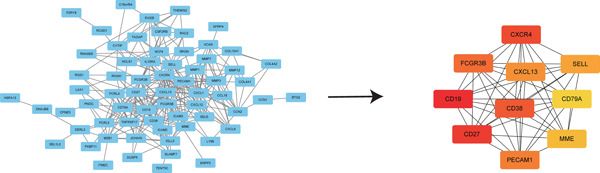
(b)

### 3.5. Construction of Forest Plot Model for Periodontitis Risk Prediction

We constructed a forest plot model based on five hub genes to predict periodontitis risk, which accurately distinguished between periodontitis patients and healthy controls. ROC curve analysis of these genes confirmed the model’s high predictive capability, evidenced by high AUC values (Figure 5a,b), with specific AUC values for CXCR4, CD19, CD27, FCGR3B, and CD79A being 0.907, 0.879, 0.886, 0.867, and 0.885, respectively.

Figure 5Risk prediction of periodontitis using forest plot. (a) Forest plot of multivariable logistic regression for the five most significant hub genes (CXCR4, CD19, CD27, FCGR3B, and CD79A). (b) ROC curves to assess the diagnostic efficacy of the forest plot model and each hub gene.

**Figure:**
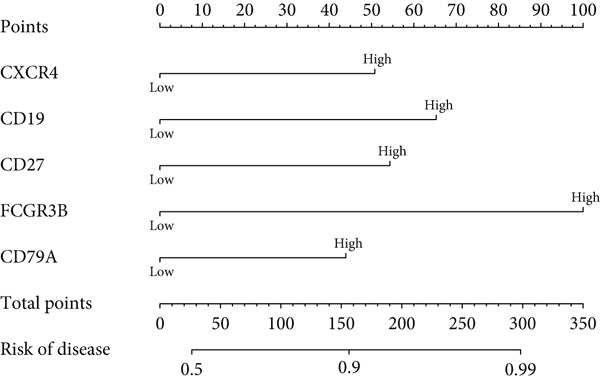
(a)

**Figure:**
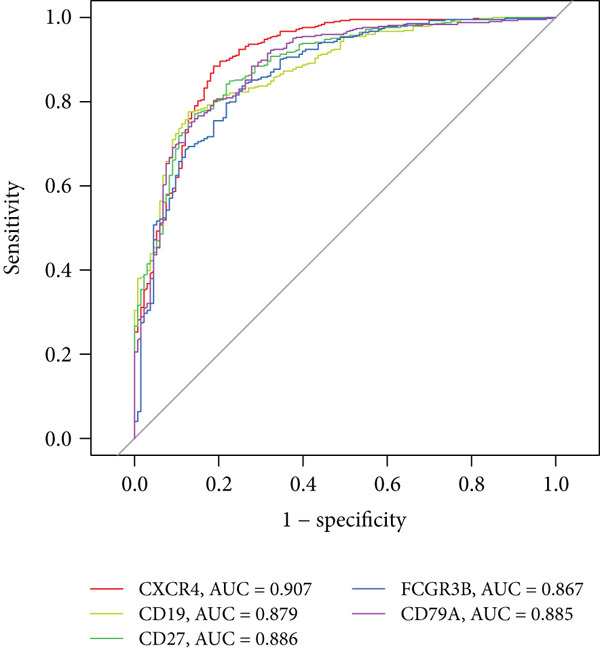
(b)

### 3.6. Causal Relationship Between CD27 and Periodontitis Risk

The results of MR illustrate the influence of each genetic variant on periodontitis. In particular, we focused on examining the connection between CD27 and periodontitis. By employing the IVW method, we discovered a causal link between CD27 and the development of periodontitis (OR = 0.7536, 95*%*CI = 0.5886–0.9647, *p* = 0.02477). Meanwhile, the MR–Egger method produced nonsignificant outcomes (OR = 0.7295, 95*%*CI = 0.4681–1.1368, *p* = 0.18147). An evaluation of the funnel plot indicated a symmetrical distribution, and the MR–Egger regression’s intercept did not reveal evidence of horizontal pleiotropy (*p* = 0.863204), confirming no inherent bias stemming from pleiotropic effects. A systematic MR analysis that excluded each SNP one by one consistently demonstrated causal significance, suggesting that no single SNP predominantly affects the relationship between CD27 levels and periodontitis. These results support the reliability of earlier MR findings (Figures 6a, 6b, 6c, and 6d). Detailed information regarding the SNP characteristics related to CD27 and periodontitis can be found in Supporting Information 3: Table S3.

Figure 6Results of Mendelian randomization study. (a) Scatter plot of SNP effects on CD27 expression (*x*‐axis, *β*‐exposure) vs. periodontitis risk (*y*‐axis, *β*‐outcome). (b) Forest plot displaying the causal effects of each SNP on the risk of periodontitis. (c) Funnel plot used to visualize the overall heterogeneity of MR estimates of CD27’s impact on periodontitis. (d) Leave‐one‐out plot to visualize the causal effect of CD27 on periodontitis risk when leaving one SNP out.

**Figure:**
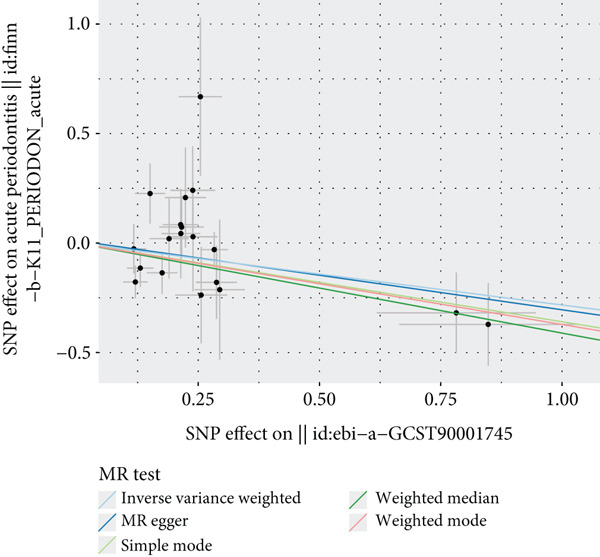
(a)

**Figure:**
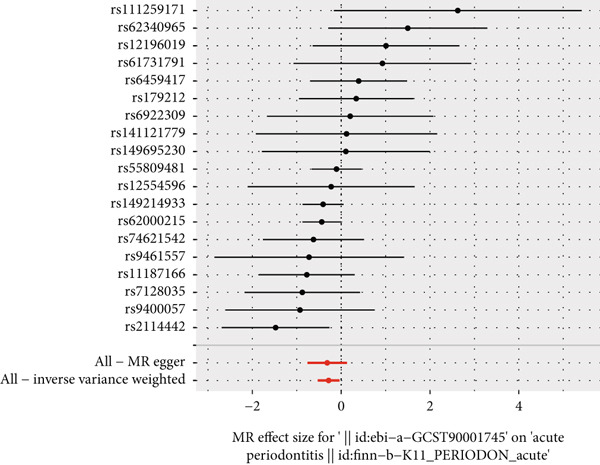
(b)

**Figure:**
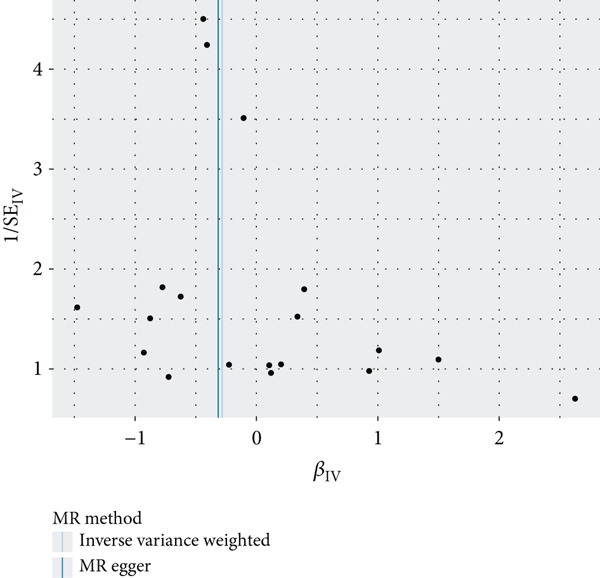
(c)

**Figure:**
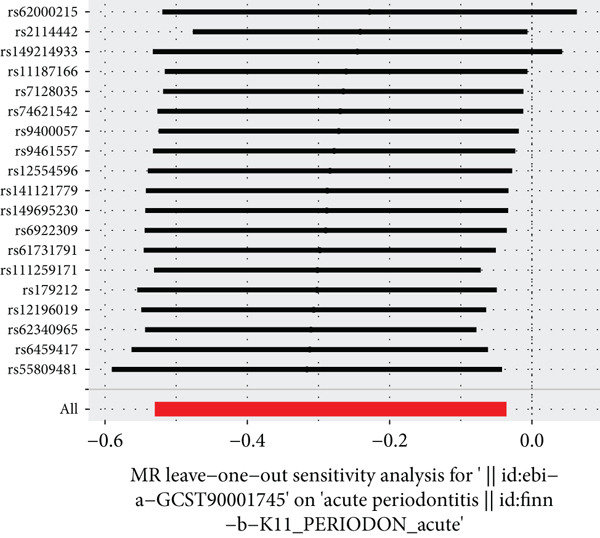
(d)

### 3.7. Assessment of Immune Cell Infiltration in Periodontitis

We examined the variations in immune cell infiltration between control samples and those affected by periodontitis (see Figure 7a,b), along with the correlation analysis of different immune cells (refer to Figure 7). The findings indicated unique patterns of immune cell infiltration linked to the expression of the key gene CD27 in individuals with periodontitis. Notably, there was a positive correlation between CD27 expression and the infiltration levels of plasma cells as well as activated CD4 memory T cells, whereas a negative correlation was observed with the infiltration levels of eosinophils, M2 macrophages, CD8 T cells, resting mast cells, and monocytes (see Figure 7d and Supporting Information 4: Figure S1).

Figure 7Assessment of immune cell infiltration in periodontitis. (a) Relative distribution heatmap of 22 immune cells in normal and periodontitis samples. (b) Violin plots comparing key cell types between groups: asterisks (∗∗) indicate significance ( ^∗^ *p* < 0.05,  ^∗∗^ *p* < 0.01, and  ^∗^ *p* < 0.001, Wilcoxon test). (c) Correlation analysis among immune cells. (d) Correlation analysis between CD27 and immune cell infiltration.

**Figure:**
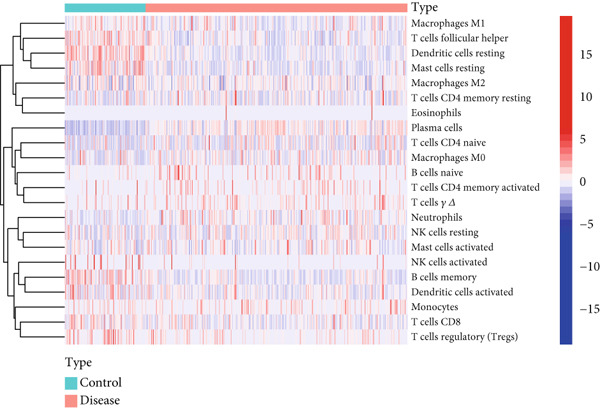
(a)

**Figure:**
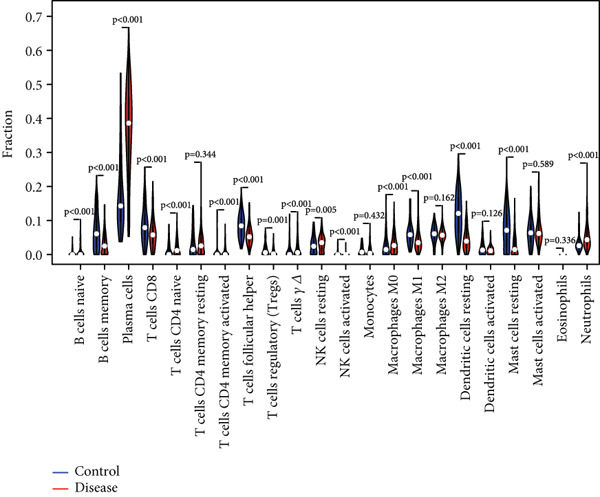
(b)

**Figure:**
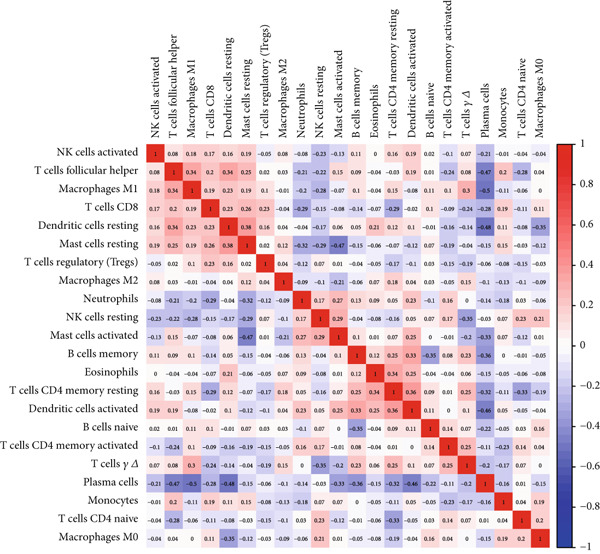
(c)

**Figure:**
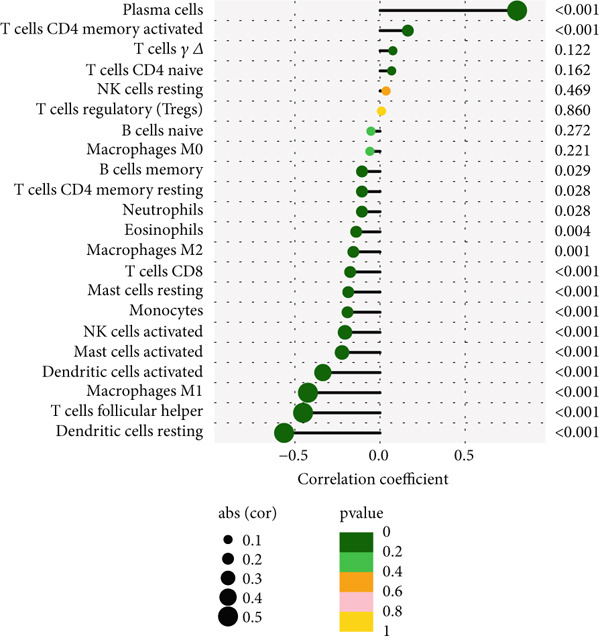
(d)

## 4. Discussion

Periodontitis, classified among the world’s major noncommunicable diseases, affects approximately 45%–50% of the global population. Its most severe form impacts around 11.2%, making it the sixth most common human ailment [1, 2]. This chronic inflammatory disease damages the soft tissues and bones around the teeth and is prevalent in adults, with severe cases occurring in up to 20% of individuals [3]. The disease’s etiology involves a complex interplay between bacterial infection and the host immune response, further influenced by individual behavioral habits and systemic health conditions [22, 23]. Pathogens such as *Porphyromonas gingivalis*, *Tannerella forsythia*, and *Treponema denticola*, found in nearly every niche of the oral cavity, complicate treatment efforts [4].

In this research, we carefully examined RNA‐seq data derived from both periodontitis and healthy samples found in the GSE16134 and GSE10334 datasets. Through the application of bioinformatics techniques, we discovered 143 genes with significant differential expression, comprising 115 that were upregulated and 28 that were downregulated. By utilizing WGCNA, we identified a central set of genes that are strongly linked to periodontitis. Integrating these differentially expressed genes with WGCNA results, we narrowed down to 98 key genes linked to periodontitis. GO and KEGG functional clustering and pathway analysis provided insights into their roles in disease onset and progression, especially in critical biological processes and signaling pathways like B cell activation, leukocyte migration, and cytokine–receptor interaction. The analysis of the PPI network shed more light on the associations between these genes, emphasizing important nodes including CXCR4, FCGR3B, CXCL13, SELL, CD19, CD38, CD79A, CD27, MME, and PECAM1. These genes, central to the network, underscore their potential roles in periodontitis’ occurrence and development.

MR analysis offers a novel perspective on exploring the genetic basis of periodontitis, particularly by addressing the confounding and reverse causality issues common in traditional association studies [24–28]. This approach allows for the investigation of polygenic or potential causal genes associated with periodontitis, opening avenues for discovering disease mechanisms and new therapeutic targets [29]. Significantly, we explored the possible causal link between the CD27 gene and periodontitis. The CD27 gene, which is part of the tumor necrosis factor receptor superfamily (TNFRSF) [30, 31], is present on CD4+ and CD8+ T lymphocytes, natural killer (NK) cells, and thymocytes, and its expression is triggered during the activation of B cells [32, 33]. Our MR analysis confirmed a causal relationship between CD27 and periodontitis, suggesting CD27 as a potential therapeutic target.

CD27’s functional significance extends beyond the costimulation of immune cells; it plays a pivotal role in immune responses, particularly in activating cytotoxic CD8+ T cells [34]. By promoting T cell proliferation, survival, and effector functions, CD27 signaling bolsters the immune system’s responsiveness, potentially enhancing resistance against periodontal pathogens [31, 35]. Moreover, CD27/CD70 costimulation may also boost the innate immune system, including interferon‐gamma production in NK cells [36].

CD27 not only acts as an immune costimulatory molecule to promote T cell activation, but its function also extends to regulating local immune imbalance and bone resorption in periodontitis. In the Th17/Treg imbalance induced by dental plaque biofilm, CD27–CD70 signaling can promote Th17 cell differentiation and inhibit Treg function, leading to increased IL‐17 levels, which in turn activate osteoclasts through the RANKL/OPG axis and accelerate alveolar bone resorption [37, 38]. In addition, CD27 signaling strengthens immune defense against periodontal pathogens by promoting CD8+ T cell proliferation, survival, and effector function. Its interaction with CD70 can also enhance interferon‐*γ* production by NK cells, further strengthening the innate immune response. Together, these mechanisms reveal the key role of CD27 in the regulation of periodontal inflammation and bone remodeling, supporting its biological feasibility as a potential diagnostic and therapeutic target.

When evaluating immune cell infiltration in periodontitis, we discovered unique patterns related to the expression levels of CD27, notably positive correlations with the infiltration of activated CD4 memory T cells and plasma cells, while negative correlations were observed with the levels of resting mast cells, eosinophils, M2 macrophages, CD8 T cells, and monocytes, among others. Our analysis of immune cell presence in periodontitis highlighted these distinct patterns linked to CD27 expression, suggesting the significant role of CD27 in regulating immune responses and its potential contribution to the progression of periodontitis.

Recent advances in AI‐driven protein structure prediction, particularly the AlphaFold model, offer a transformative avenue to bridge our gene‐level findings with mechanistic and therapeutic development [39]. By generating high‐resolution three‐dimensional structures for CD27, CXCR4, and other hub proteins, AlphaFold enables the identification of key ligand‐binding interfaces—such as the CD27–CD70 interaction site—underlying immune signaling in periodontitis. Moreover, AlphaFold‐Multimer can simulate the assembly of multiprotein complexes (e.g., CD27–CXCR4–CD19), shedding light on synergistic mechanisms that drive local inflammation and bone resorption. Integrating GWAS‐identified SNPs into these structural models further permits in silico assessment of variant‐induced conformational changes and their impact on binding affinities. Such insights can directly inform molecular docking screens for small‐molecule inhibitors or monoclonal antibodies aimed at disrupting pathogenic PPIs, thereby accelerating the translation from causal gene discovery to precision therapeutic design.

Although this study is the first to reveal a genetic causal relationship between CD27 and periodontitis through bioinformatics and MR analyses—offering new avenues for diagnosis and treatment—it has several limitations. It relies on public datasets (GSE16134, GSE10334) and lacks experimental validation of CD27 expression and function in periodontal tissues; sample size and geographic representation are limited, and the scarcity and incomplete clinical annotation of available data prevented external cohort validation, potentially restricting the model’s generalizability across populations and settings. Although we applied stringent SNP selection (*p* < 5 × 10^−8^, *r* ^2^ < 0.01) and performed multiple sensitivity analyses, MR estimates may still be affected by weak‐instrument bias, horizontal pleiotropy, and gene–environment interactions. In future work, we will validate the role of CD27 in inflammatory cell infiltration and bone remodeling using in vitro cell culture and in vivo animal models and explore the CD27–CD70 axis and downstream mechanisms. We also plan to recruit multicenter, prospective clinical cohorts from diverse regions and populations for high‐quality transcriptomic sequencing, conduct external validation and parameter optimization of our model, and leverage larger GWAS datasets with stronger instruments to bolster MR reliability and fully characterize CD27’s expression and functional dynamics at different stages of periodontitis. Additionally, we will employ AlphaFold‐Multimer to simulate the assembly of multiprotein complexes, such as CD27–CXCR4–CD19, to elucidate the synergistic mechanisms driving local inflammation and bone resorption.

## 5. Conclusion

By leveraging WGCNA alongside MR methods, this study has effectively identified key genes intimately associated with periodontitis, thereby unveiling novel insights into the complex genetic and molecular mechanisms underlying the disease. Significantly, it clarifies the cause‐and‐effect relationship linking the CD27 gene to the development of periodontitis, thereby presenting new possible targets for preventing and treating the disease. Additionally, through analyzing the variations in immune cell infiltration among individuals with periodontitis compared to those who are healthy, our study enhances the comprehension of the immunoregulatory processes involved in periodontitis and emphasizes the importance of certain immune cells in its advancement. In summary, by uncovering the genetic and immunological mechanisms behind periodontitis, we pave the way for potential applications in its prevention, diagnosis, and treatment.

## Disclosure

All authors read and approved the final manuscript.

## Conflicts of Interest

The authors declare no conflicts of interest.

## Author Contributions

K.X. designed the study, conducted the data analysis, conceptualized the research framework, guided the overall methodology, and drafted the manuscript. C.L. handled data collection and performed the initial analysis. N.H. handled data collection, guided the overall methodology, and finalized the manuscript revisions.

## Funding

The study is supported by the Chongqing Medical Scientific Research Project (2015MSXM048).

## Supporting Information

Additional supporting information can be found online in the Supporting Information section.

## Supporting information


**Supporting Information 1** Table S1. Differentially expressed genes (DEGs) between the periodontitis and control groups (GSE16134, GSE10334).

**Supporting Information 2** Table S2. Gene modules identified through WGCNA in GSE16134 and GSE10334.

**Supporting Information 3** Table S3. SNP characteristics related to CD27 and periodontitis, with causal effect results from Mendelian randomization.

**Supporting Information 4** Figure S1. Correlation analysis of CD27 expression and immune cell infiltration in periodontitis samples.

## Data Availability

The datasets generated in this study are available in the online NCBI GEO repositories at the following links: https://www.ncbi.nlm.nih.gov/geo/query/acc.cgi?acc=GSE16134 and https://www.ncbi.nlm.nih.gov/geo/query/acc.cgi?acc=GSE10334.
