# Supplementary figures and images for: Identification of Key Periodontitis Genes and Their Mechanisms of Action Using Comprehensive Multiple Microarray Analysis and Mendelian Randomization Methods
Source: Int J Genomics. 2025 Oct 23;2025:5587468. doi: 10.1155/ijog/5587468 (PMC12548494; doi:10.1155/ijog/5587468)

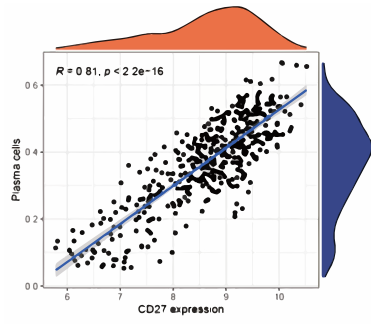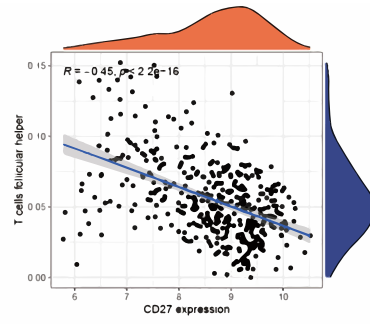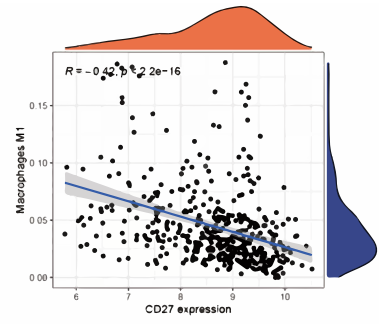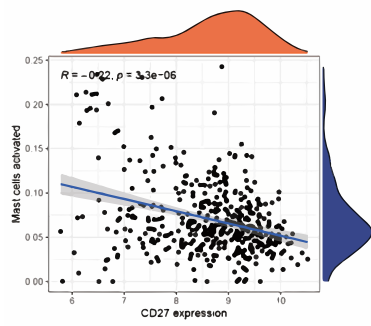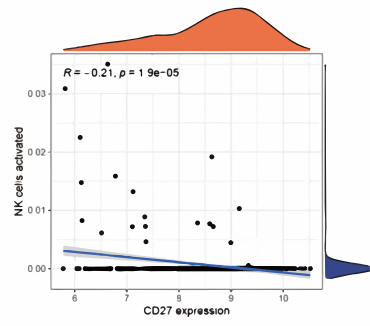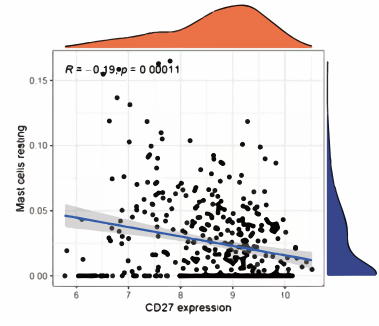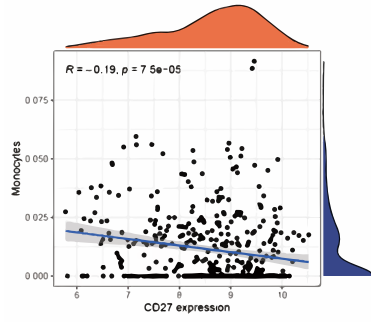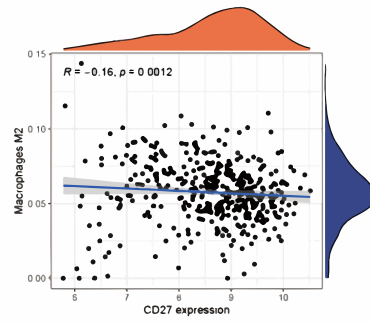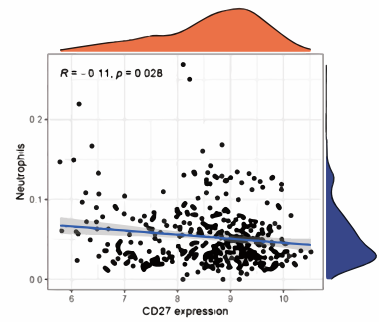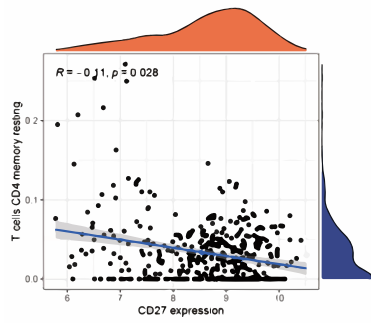

Supplement: Supplementary file 4 — Supporting Information 4 Figure S1. Correlation analysis of CD27 expression and immune cell infiltration in periodontitis samples. [file IJOG-2025-5587468-s004.pdf]
